# Comprehensive Nutrient Gap Assessment (CONGA): A method for identifying the public health significance of nutrient gaps

**DOI:** 10.1093/nutrit/nuaa140

**Published:** 2021-03-08

**Authors:** Ty Beal, Jessica M White, Joanne E Arsenault, Harriet Okronipa, Guy-Marino Hinnouho, Saul S Morris

**Affiliations:** 1 Global Alliance for Improved Nutrition, Washington, DC, USA; 2 Department of Environmental Science and Policy, University of California, Davis, California, USA; 3 United Nations Children's Fund, New York, New York, USA; 4 Institute for Global Nutrition, University of California, Davis, Davis, California, USA; 5 Intake, Center for Dietary Assessment, FHI Solutions, Washington, DC, USA; 6 Institute for Global Nutrition, University of California , Davis, Davis, California, USA; 7 Department of Population Medicine and Diagnostic Sciences, Cornell University, Ithaca, New York, USA; 8 Institute for Global Nutrition , University of California , Davis, Davis, California, USA; 9 Helen Keller International, New York, New York, USA; 10 Global Alliance for Improved Nutrition, London, UK

**Keywords:** dietary intake, food consumption, micronutrient deficiencies, nutrient adequacy, nutrient gap assessment

## Abstract

Identifying dietary nutrient gaps and interpreting their public health significance are essential for improving poor diets and reducing malnutrition. Evidence indicative of the burden of nutrient deficiencies and inadequate nutrient intake or availability exists in many countries yet is often misinterpreted or underused in decision-making. Clear guidance is lacking on how to synthesize and interpret the relevant evidence, which comes in many forms. To fill this methodological gap, an approach called Comprehensive Nutrient Gap Assessment was created to enable use of existing evidence to assess the public health significance of nutrient gaps and identify evidence gaps. Comprehensive Nutrient Gap Assessment requires ≥ 2 experts in nutritional assessment but does not require primary data collection or secondary quantitative data analysis. It can be implemented relatively quickly with low costs, for specific countries and subnational regions, and updated on the basis of new data with minimal effort. The findings from a Comprehensive Nutrient Gap Assessment are easily interpretable by nontechnical decision makers yet include clear justification for technical audiences.

## INTRODUCTION

Dietary nutrient gaps represent specific nutrient shortfalls in the diet that can lead to deficiency and poor health. Yet, identifying dietary nutrient gaps and interpreting their public health significance are challenging, even for specialists. The preferred evidence on nutrient gaps is based on biological, clinical, or functional markers and nutrient adequacy of individual diets. However, there is limited guidance on assigning public health significance from prevalence ranges for many commonly available biomarkers^1^ or indicators of inadequate nutrient intakes. Moreover, these robust types of evidence often are unavailable or have small sample sizes, lack recent data, and/or provide limited representation of the geography or age and sex group of interest. Less robust types of evidence, such as nutrient adequacy of national food supplies^2^ or nutrient-informative food-group intake of individuals (eg, iron-rich food intake among young children) or households (eg, household salt iodization), often exist where more robust types of evidence do not, may have larger sample sizes based on more recent data, and/or may offer better geographic, age, or sex representation.

The aim of this study was to develop a method to synthesize and interpret existing evidence informative of nutrient gaps, which comes from disparate sources of varying robustness and representation. This new approach, called Comprehensive Nutrient Gap Assessment (CONGA), was developed on the basis of theory of health implications of nutrient deficiencies at the individual and population levels and knowledge of the utility and quality of biomarkers and dietary data.^3^ Comprehensive Nutrient Gap Assessment can be used to identify nutrient gaps and their public health significance among target populations for the purpose of informing policies and programs aimed at improving nutrient adequacy and reducing undernutrition. Specifically, CONGA allows for better targeting and prioritization of micronutrients with the most significant gaps as well as identification of the quality of evidence that policies or programs may be based on. For illustrative purposes, this article and 2 related articles in this supplement of *Nutrition Reviews* that apply the CONGA method^4^^,^^5^ focus on 11 micronutrients commonly lacking in the diets of young children in low- and middle-income countries.^6^ The CONGA method can also be extended to other nutrients and age, sex, and physiological groups, depending on data availability.

We begin by describing types of evidence recommended to inform nutrient gaps and discuss their strengths and limitations. We then present the CONGA method in detail, including how to assess the burden of nutrient gaps and certainty of evidence. We conclude by highlighting the unique utility of CONGA and implications for decision makers and future research.

## EVIDENCE TYPES INFORMATIVE OF NUTRIENT GAPS

Comprehensively assessing nutrient gaps requires understanding and use of disparate evidence types. Table 1 outlines 5 evidence types that can be used to inform nutrient gaps. In this section, we discuss the considerations for using each evidence type to assess nutrient gaps and provide the background theory underlying the CONGA method described in the next section.

**Table 1:** Five main evidence types for assessing nutrient gaps

Evidence type	Data type	Strengths	Limitations
Biological, clinical, and functional markers	Blood tests Urine tests Physical examination	Direct marker of individual physiological status Moderately easy to understand morbidity and mortality risk burden Accounts for poor bioavailability and/or absorption	Influenced by nondietary factors (eg, diseases that cause malabsorption or blood loss) Not widely available nationally for most nutrients and populations
Nutrient adequacy of individual diets (modeled using food composition and requirement data)	Quantitative dietary intake (eg, weighed records or 24-hour recall) Semiquantitative food frequency questionnaire (ie, includes some estimate of portion sizes)	Direct marker of individual dietary intake	Wide variation in quality of data and modeling approach Not widely available nationally for many nutrients and populations Somewhat difficult to understand morbidity burden
Nutrient adequacy of household diets (modeled using food composition and requirement data)	Quantitative dietary intake (eg, household intake) Semiquantitative food frequency questionnaire (ie, includes some estimate of portion sizes)	Nationally representative and frequently available for many low- and middle-income countries	Wide variation in quality of data and modeling approach Does not directly measure individual intake (must estimate using adult-equivalent method) Limited specificity of foods (poor match with food composition data) Somewhat difficult to understand morbidity burden
Nutrient adequacy of national food supplies (modeled using food composition and requirement data)	Food supply data (ie, Food and Agriculture Organization food balance sheets)	Standardized, nationally representative of the entire population, and available for nearly every country annually since 1961	Does not measure intake at any level Does not adequately account for home or small-scale production, wild harvest, or household food waste Limited specificity of foods (poor match with food composition data) Difficult to properly account for fortification Difficult to understand morbidity burden
Nutrient-informative food-group intake of individuals or households	Quantitative dietary intake (eg, weighed records, 24-hour recall, or household intake) Semiquantitative food frequency questionnaire (ie, includes some estimate of portion sizes) Nonquantitative food frequency questionnaire (eg, prevalence of intake in past 24 hours or households with iodized salt)	Frequently available nationally for many populations	Only useful for certain nutrients (eg, iron- or vitamin A–rich foods, iodized salt) Quality of salt iodization is not always tested; does not account for food sources of iodine, which vary substantially geographically Difficult to understand morbidity burden

### Biological, clinical, and functional markers

Biomarkers, including those detectable in blood and urine samples, and clinical or functional markers based on physical examination provide an indication of prevalence of nutrient deficiencies. There are 6 sentinel micronutrients for which biomarkers are commonly collected in population-based surveys and of which deficiency indicates risk of severe and/or long-term consequences: iron, vitamin A, iodine, zinc, folate, and vitamin B_12_. Iron deficiency is a primary cause of anemia and can result in cognitive impairment, decreased work productivity, and death.^7^ Vitamin A deficiency has severe consequences, even with mild deficiency, including night blindness, increased susceptibility to infections, and death.^8^ Iodine deficiency has severe consequences, even with mild or moderate deficiency, including growth and cognitive impairment, goiter, and death, often due to deficiency during pregnancy.^9^ Zinc deficiency in children is associated with poor health, increased risk of diarrhea, and impaired cognitive and motor development.^10^^,^^11^ Vitamin B_12_ deficiency in infants has immediate and long-term consequences, including anemia, developmental regression, and depression during adulthood, and affects cognitive outcomes in adulthood.^12^^,^^13^ Finally, folate deficiency in infants and young children can have immediate and long-term consequences, including anemia, hindered brain development, and adult depression, and in pregnant women can cause neural tube defects in the fetus.^12^

Individual biological, clinical, and functional markers can be difficult to interpret alone and require careful consideration of other biomarkers and infection and inflammation burden. Some nutrients have multiple biomarkers to indicate nutrient deficiency in populations, which can result in considerable differences in prevalence estimates.^14^^,^^15^ For example, iron status of populations can be assessed by 1 or multiple indicators, including serum ferritin and serum iron levels, total iron-binding capacity, transferrin saturation percent, erythrocyte protoporphyrin, soluble transferrin receptor, and the ratio of soluble transferrin receptor to serum ferritin.^16^ Although established cutoffs for deficiency or severity of deficiency exist for many biomarkers, there are some discrepancies, and some vary depending on the assay manufacturer. Moreover, large-scale nutrition surveys sometimes use different cutoffs than those recommended by scientific bodies. Furthermore, some surveys do not adjust for infection or inflammation, which can lead to under- or overestimates of deficiency prevalence depending on the biomarker, particularly serum retinol and serum ferritin.^14^^,^^15^

The severity of health consequences from deficiency also varies by nutrient and cutoff, and it is difficult to understand the magnitude of dietary nutrient gaps from biomarkers, because nondietary factors also influence nutrient status. For example, an individual may consume adequate bioavailable iron but iron deficiency still may develop from pathological malabsorption or blood loss.^16^ There is guidance on the public health significance of prevalence ranges for deficiencies of several nutrients, including vitamin A,^17^ iodine, thiamine, niacin, and vitamin C.^1^ However, guidance on iodine deficiency is challenging because of within-person variation in urinary iodine concentration (UIC),^18^ the most commonly available iodine biomarker. For this reason, the World Health Organization (WHO) recommends using median UIC to assess iodine status of populations. However, based on the current guidance, a population with a median UIC of 100 μg/L would be considered iodine sufficient, while at the same time, half of the population could be at risk for mild deficiency. And even mild-to-moderate iodine deficiency can hinder child growth and cognitive development and cause diffuse goiter.^9^ The guidance on the public health significance of prevalence ranges for thiamine, niacin, and vitamin C deficiencies are rarely applicable, because biomarkers for these nutrients are not usually available at the national level. To our knowledge, there is no global guidance on the public health significance of different prevalence ranges for iron, zinc, folate, vitamin B_12_, calcium, or vitamin D deficiencies.

Specific guidance on the public health significance of anemia prevalence exists.^19^ Biomarkers that singularly assess anemia status through hemoglobin, hematocrit, or red blood cell count have been used to inform estimates of prevalence of iron deficiency. However, the proportion of anemia due to iron deficiency varies substantially by country and population, even among countries with similar infectious-disease burdens.^7^ Thus, any attempt to estimate iron deficiency prevalence or burden from anemia prevalence must be done with supporting evidence of the causes of anemia in the population of interest. In addition, the sampling method (venous or capillary), analytical method, and assay manufacturer can influence prevalence estimates of anemia.^20^^,^^21^

To a lesser extent, clinical or functional markers assessed via physical examination are sometimes used to indicate the prevalence and burden of micronutrient deficiencies at the population level. Common indicators include goiter for iodine deficiency and Bitot spots or night blindness for vitamin A deficiency.^1^ Clinical and functional markers typically have high specificity but low sensitivity for the underlying deficiency. For example, there is a high probability that iodine or vitamin A deficiencies detected by goiter or Bitot spots, respectively, will represent true deficiencies, because these markers typically represent specific physical consequences of deficiency; however, there is also a high probability that a considerable proportion of the individuals assessed will be misdiagnosed with no deficiency, when, in reality, they are deficient or have depleted status but have not yet developed visible signs as a result.

### Nutrient adequacy of individual diets

Modeling studies that combine data on individual dietary intake, food composition, and nutrient requirements can estimate the prevalence of inadequate nutrient intakes for a given population. The most common approach is the estimated average requirement (EAR) cut-point method, which calculates the proportion of the population with intakes below the EAR—the amount required to meet the needs of 50% of the population.^22^ The EAR cut-point method can be used when the distribution of nutrient requirements is normally distributed, and it is valid when intakes and requirements are independent, the distribution of intakes varies more than the distribution of requirements, and the true prevalence of inadequate intakes is not very high (≥ 90%) or very low (≤ 10%).^23^ Violating these assumptions can over- or underestimate the true prevalence of inadequate intakes to varying degrees.^23^ In instances where the distribution of requirements is not normally distributed, such as iron requirements for menstruating women, the probability approach should be used.^24^

The data collection method used to assess quantitative dietary intakes can substantially influence the accuracy of prevalence estimates. The gold standard is a weighed-food record, in which participants or enumerators weigh all foods and beverages at the time of consumption.^25^ However, weighed-food records are typically only conducted for small samples that are not nationally representative. More common and still high-quality are 24-hour dietary recalls, in which enumerators probe respondents about all foods and beverages consumed in the previous 24 hours.^26^ Also common but typically less accurate for estimating nutrient intakes are semiquantitative food frequency questionnaires, in which respondents recall the frequency of consuming items from a predefined food and beverage list with standard portion sizes over a specified period.^27^ Even within these 3 approaches, the quality of specific surveys can vary substantially and must be critically reviewed when considering the certainty of the estimates. For 24-hour recalls, data from at least 2 nonconsecutive days of intake are typically required for a subset of individuals to properly estimate the population distribution of usual nutrient intakes. Also, the season during which dietary surveys are administered can significantly influence estimated nutrient intakes, particularly from foods that are only seasonally available.

Another important factor that influences the accuracy of prevalence of inadequate nutrient intake estimates is the quality of food-composition data used and the appropriateness of the match with foods identified in dietary surveys. Local (country-specific) food composition tables are often unavailable, of low quality, dated, borrow data from other sources, and/or exclude foods in the form they are typically consumed. Higher-quality food-composition databases often do not contain all the specific foods found in different individual countries. Regardless of the food composition database or databases used, foods are not always appropriately matched with foods in the dietary data, which can lead to additional inaccuracies.

Finally, there are multiple sources of nutrient requirement data and they report different values depending on the nutrient and age and sex group. For instance, the United Nations’ Food and Agriculture Organization (FAO)/WHO recommended nutrient intakes^28^ provide 3 values for zinc and 4 for iron, depending on bioavailability, whereas the Institute of Medicine dietary reference intakes recommendations^29^ do not, so differences between zinc and iron requirements can range in orders of magnitude depending on which bioavailability category is chosen. Some countries have their own EARs, so it is important to pay attention to which nutrient requirement data are used (and any differences) when comparing results from different studies. Widespread adoption of globally harmonized nutrient reference values would allow for more consistent nutrient adequacy estimates.^30^ Importantly, researchers often mistakenly use recommended daily allowances (RDAs) or recommended nutrient intakes instead of EARs to calculate the prevalence of inadequate intakes, which results in overestimates.

### Nutrient adequacy of household diets

Modeling studies can also use household food consumption and adult-equivalent methods, which assume foods are distributed within a household according to its members’ energy and/or nutrient requirements, to estimate the prevalence of inadequate nutrient intakes for a given population.^31^ Household consumption is available in some household consumption and expenditure surveys (HCES), also referred to as household income and expenditure surveys, living standard measurements surveys, and household budget surveys. Nutrient adequacy modeling approaches using household consumption data are generally not as accurate or precise as those using individual dietary intake data, particularly for young children.^31^^,^^32^ However, HCES are generally nationally and subnationally representative and collected frequently in low- and middle-income countries. To date, few studies have used HCES to estimate prevalence of inadequate intakes. The methods and data sources of any modeling study using household-level data should be scrutinized because many household surveys only measure food acquisition rather than actual consumption. Moreover, the survey food lists may be limited and lack specificity, which causes poor matches with food composition data.

### Nutrient adequacy of national food supplies

A crude marker of nutrient adequacy among a country’s total population can be achieved by modeling hypothetical intake using national food balance sheets (FBS) from the FAO. This type of estimate assesses the nutrient adequacy of national food supplies, or food available for consumption, by comparing the average quantity of each nutrient available for each country to the average requirements in each country. Existing analyses using this approach have provided estimates for several micronutrients in nearly every country^2^; however, at this point, they have not been updated with the most recent FAO data, which, at the time of writing, exist for up to year 2018 (http://www.fao.org/faostat/en/#data/FBS). Food balance sheets are limited in that they do not adequately account for home or small-scale production, wild harvest, or household food waste. Furthermore, similar to HCES, FBS have limited specificity of food groups, making it difficult to appropriately match with food composition data. Importantly, food supply studies should be scrutinized for how they incorporate fortification amounts and coverage (eg, through the Global Fortification Data Exchange) because this can have a substantial impact on prevalence estimates in some countries. Despite these limitations, FBS have important strengths: They are a proxy for diets of entire national populations and are available for nearly every country annually since 1961.

### Nutrient-informative food-group intake of individuals or households

Although nutrient-adequacy modeling studies are quantitatively more informative of nutrient gaps, quantitative or nonquantitative data on individual or household intake of nutrient-informative food groups can provide some insight. Nutrient-informative food groups could refer to a fortified source of 1 nutrient (eg, household salt iodization) or intentionally categorized to include all good sources of a particular nutrient (eg, vitamin A–rich foods or iron-rich foods). Nutrient-informative food-group intake data are often nationally representative because they are routinely collected in national nutrition surveys or global survey mechanisms like the Demographic and Health Surveys, particularly for children aged 6–23 months. Depending on the survey, either a list-based or open recall of the foods consumed in the previous 24 hours is used to determine if a food group was consumed, but these methods usually do not capture the quantity consumed. These indicators can help identify potential nutrient gaps and compare them across countries.

### Other evidence

There is evidence that does not fit into any of the 5 main evidence types discussed thus far but nevertheless may still be informative of nutrient gaps. Such evidence includes intake of food groups that are not the only source of, but are high in, ≥1 nutrients (eg, dairy is high in calcium, flesh foods are high in zinc, heme-rich foods are high in bioavailable iron, and fortified complementary foods are high in multiple micronutrients); individual micronutrient supplementation coverage (eg, vitamin A or iron); linear programming studies that identify problem nutrients but do not estimate the prevalence of inadequate intake (eg, cost-of-the-diet analyses and some Optifood analyses); and other biochemical, clinical, or functional markers that are informative but not directly indicative of deficiency (eg, anemia). This evidence is often more difficult to interpret, especially by nonspecialists, than the 5 clearly defined evidence types previously discussed and requires critical assessment and/or triangulation with other data points to be useful.

## COMPREHENSIVE NUTRIENT GAP ASSESSMENT

The CONGA method provides guidance on how to identify, interpret, and synthesize the evidence types informative of nutrient gaps identified in Table 1 and previously detailed in this article. The method also explicitly considers and accounts for differences in robustness and representativeness of different data. In this section, we describe the 8 methodological steps included in CONGA. At least 2 specialists with expertise in nutritional assessment are required to complete a CONGA. A template to complete each step of a CONGA is available in Table S1 in the Supporting Information online. Examples of applying the CONGA method in Eastern and Southern Africa and South Asia can be found in other articles in this journal supplement.^4^^,^^5^

### Step 1: Identify and compile relevant evidence and metadata

Before beginning a CONGA, decide on the geographic area(s) of interest (eg, subnational region or country), the target age and sex group, and the nutrients to assess. Although a CONGA can be conducted for any population and nutrient, the results will be less useful if severely limited by data availability. After conducting 14 CONGAs for children aged 6–23 months in Eastern and Southern Africa^4^ and South Asia,^5^ robust evidence was often available for iron and vitamin A, and, to a lesser extent, for iodine, zinc, vitamin B_12_, folate, vitamin D, and calcium, but typically not for other nutrients.

Next, decide on a search approach and method of resource (ie, literature) acquisition. The search method can be systematic or nonsystematic, depending on available time and resources, but should include grey literature because many relevant evidence sources are not in peer-reviewed journals. PubMed is the most relevant database for systematic searches, because it contains > 30 million biomedical literature citations, covering all evidence types discussed in the previous section of this article. However, it does not contain all relevant literature published in journals from low- and middle-income countries. If conducting a nonsystematic search in PubMed or Google Scholar, sorting by best match or relevance will increase the likelihood of capturing the most relevant sources. Grey literature can be identified by searching Google and Google Scholar, browsing relevant global survey repositories (eg, Demographic and Health Surveys), and consulting in-country stakeholders. For this phase, consider excluding studies in which data collection concluded > 20 years ago, with small sample sizes (n < 50), with limited geographic representation (< 10% of the total population of the geographic area of interest), and highly vulnerable participants (eg, hospitalized patients), unless they are the target population.

Compile findings and characterize the relevant evidence sources in a summary spreadsheet to aid synthesis. The spreadsheet should include columns to summarize each data point (eg, point estimate, indicator type, and cutoff, if applicable) and its metadata, as outlined in Table 2 (evidence type, geographic representation, recency of data collection, age and sex representation, and sample size). In addition, create columns to note the source and any comments on methodological or data-quality issues and temporal trends. This spreadsheet will be used in the following steps to complete the ratings.

**Table 2:** Suggested metadata and weights^a^

Evidence type	Geographic representation^b^	Recency of data collection	Age and sex representation	Sample size
Biological, clinical, and functional markers (5) Nutrient adequacy of individual diets (3) Nutrient adequacy of household diets (2)^c^ Nutrient adequacy of national food supplies (1) Nutrient-informative food-group intake of individuals or households (1)	Representative of the entire geographic area of interest (5) Representative of 75%–99% of the total population in the geographic area of interest (4) Representative of 50%–74% of the total population in the geographic area of interest (3) Representative of 25%–49% of the total population in the geographic area of interest (2) Representative of 10%–24% of the total population in the geographic area of interest (1)	< 3 y (5) 3–4 y (4) 5–6 y (3) 7–8 y (2) 9–10 y (1)	Estimates for exact age and sex group of interest (5) Estimates for either a subgroup within the age and sex group of interest representing at least half of the group; or an age and sex group, at least half of which includes the age and sex group of interest (4) Estimates for an age and sex group that includes the age and sex group of interest, but less than half of which includes the age and sex group of interest; or estimates for an age and sex group that is similar to but excludes the age and sex group of interest entirely (3) Household or food balance sheet estimates, less than half of which includes the age and sex group of interest (2) Estimates for an age and sex group that is similar to but excludes the age and sex group of interest entirely (1)	> 1,000 (5) 500–1,000 (4) 300–499 (3) 100–299 (2) 50–99 (1) Based on national food supplies (0)

a Numbers in parentheses represent the weight value to be used in weight-score calculations.

b Divide the population total that the study is representative of by the total population of the geographic area of interest.

c If the population of interest is very young children, this score should be reduced to 1.

Each row of the spreadsheet should represent a single nutrient-specific data point. If there are multiple data points for a single evidence type collected using the same survey methodology but for different years (eg, 2 national estimates of vitamin A deficiency among children aged 6–59 months from Demographic and Health Surveys in the past 10 years), include the most recent data point as a row and the older data point information in the comment column. However, if there are multiple data points for a single evidence type collected using different survey methodology (eg, 1 subnational vitamin A–deficiency estimate for children aged 6–35 months from a program-specific survey and 1 national estimate of vitamin A deficiency in children aged 6–59 months from a Demographic and Health Survey), these should each be represented in their own row.

### Step 2: Rate implied nutrient gap burden scores

After compiling all relevant evidence and metadata, assign an implied nutrient gap burden score (ie, negligible, low, moderate, or high) for each data point, following the suggested prevalence and mean ranges for commonly available population-level indicators from all 5 evidence types outlined in Table 3.

**Table 3:** Suggested prevalence/mean ranges for implied nutrient gap burden scores^a^

		Implied nutrient gap burden score^b^			
Biomarker	Age	Negligible	Low	Moderate	High
Iron^c^					
Serum ferritin (< 12 µg/L)^d^	< 5 y^e^	< 3	3–9	10–19	≥ 20
Vitamin A					
Serum retinol (< 0.7 µmol/L)^f^	6–71 m	< 3	3–9	10–19	≥ 20
Zinc					
Serum zinc (< 9.9 µmol/L)^g^	< 10 y	< 3	3–9	10–19	≥ 20
Folate					
Serum folate (< 10 nmol/L)^h^	All	< 3	3–9	10–19	≥ 20
Vitamin B_12_					
Plasma B_12_ (< 150 pmol/L)^h^	All	< 3	3–9	10–19	≥ 20
Iodine					
UIC (< 100 µg/L)	≥ 6 y^i^	< 25	25–49	50–74	≥ 75
Median UIC (µg/L)^j^	≥ 6 y	≥ 150	100–150	50–99	< 50
Total goiter rate^j^	6–12 y	< 3	3–9	10–19	≥ 20
Inadequate intake or availability					
Iron, vitamin A, zinc, folate, vitamin B_12_	All	< 5	5–14	15–24	≥ 25
Calcium, niacin, thiamine, vitamin B_6_	All	< 5	5–19	20–49	≥ 50
Vitamin C	All	< 10	10–29	30–49	≥ 50
Nutrient-informative food group intake					
Vitamin A-rich foods (past 24 hours)	6–23 m	> 90	75–90	60–74	< 60
Iron-rich foods (past 24 hours)	6–23 m	> 70	60–70	50–59	< 50
Household iodized salt coverage	All	> 90	75–90	60–74	< 60
Household adequately iodized salt coverage (≥ 15 ppm)	All	> 80	65–80	50–64	< 50

*Abbreviation:* UIC, urinary iodine concentration.

a These ranges may need to be adjusted depending on the indicator, level of inflammation in the population, whether adjustments were made for inflammation, and if so, the method of adjustment. Indicators not listed here will need to be assessed by an expert in nutritional assessment to estimate prevalence ranges for the implied burden score.

b Data reported as % except for Median UIC.

c If only iron deficiency anemia data are available, use similar or lower prevalence ranges as those for iron deficiency.

d Various adjustments for inflammation have differing impacts on iron deficiency prevalence.^15^ The World Health Organization (WHO) suggests iron deficiency is not prevalent when < 10% of the population is above the manufacture’s cutoff for soluble transferrin receptor values, even in populations with deficiency ≥ 20% when measured by serum ferritin.^33^

e Cutoff is < 15 μg/L for individuals > 5 years old.

f Prevalence ranges coincide with WHO recommendations.^17^ Various adjustments for inflammation have different impacts on vitamin A deficiency prevalence. Unadjusted estimates are typically 11–18 percentage points higher than adjusted estimates in areas with high inflammation.^14^ Retinol binding protein is often used as surrogate for serum retinol, which may not always be appropriate, depending on the population.

g Morning, nonfasting. Cutoff is 8.7 μmol/L in the afternoon for nonfasting children.^34^

h See de Benoist.^35^

i Excluding pregnant and lactating women.

j To align cutoffs with comparable severity across indicators, we suggested different ranges than those in the WHO recommendations.^1^ These ranges are not intended to replace WHO guidance on public health severity of iodine deficiency.

Of the indicators in Table 3, only prevalence of serum retinol < 0.70 µmol/L,^17^ median urinary iodine concentration, and total goiter rate have established guidance on public health significance.^1^ The WHO-recommended prevalence ranges for the public health significance of vitamin A deficiency (defined as serum retinol level < 0.70 µmol/l^17^) were used and applied to the commonly available biomarkers for iron, zinc, folate, and vitamin B_12_. For median urinary iodine concentration and total goiter rate, however, different ranges from WHO recommendations^1^ are suggested that better align with the severity of the indicative cutoffs across indicators. These ranges are not intended to replace WHO guidance on the public health severity of iodine deficiency.

For nonbiomarkers, prevalence ranges were used for which the resulting nutrient gap burden ratings generally corresponded with the burden ratings that were based on biomarkers from CONGAs for children aged 6–23 months from 14 countries in Eastern and Southern Africa and South Asia.^4^^,^^5^ To address the sense of using these prevalence ranges, the agreement between biomarker- and nonbiomarker-implied nutrient gap burden ratings for children aged 6–23 months in these 14 countries that qualified for the quantitative nutrient gap burden score (see step 4) was assessed (Figure S1 in the Supporting Information online). Overall, there was moderate agreement between biomarker and nonbiomarker ratings—the weighted κ (κ_w_) value, which uses linear weights to take into account how far apart the ratings are, was 0.44. There was only slight agreement for data points on nutrient adequacy of national food supplies (κ_w_ = 0.15), largely due to the small variation in ratings, moderate agreement for data points on nutrient-informative food groups of individuals or households (κ_w_ = 0.54), and insufficient data to calculate agreement for nutrient adequacy of individual or household diets. When stratifying by nutrient overall, less agreement was found, largely due to the small variation in ratings: iron (κ_w_ = 0.32), vitamin A (κ_w_ = 0.01), iodine (κ_w_ = 0.29), and zinc (κ_w_ = 0.00). In addition to the small variation in ratings, the imperfect correspondence between biomarker and nonbiomarker ratings could be due to the data having differing characteristics that can explain the differences (eg, different geographic and/or age, sex, or physiological representation, sample size, and/or recency), nondietary factors contributing to deficiency (eg, malabsorption), and/or issues with the validity of the nonbiomarker data and/or biomarker data.

The prevalence of each data point should not necessarily be taken at face value but critically assessed to determine to what extent it may be over- or understating the true prevalence and burden. For example, the prevalence ranges for biomarkers in this table may need to be adjusted depending on the indicator, assay type and manufacturer; level of inflammation in the population of interest; and whether adjustments were made for inflammation, and if so, the method of adjustment. The ranges for prevalence of inadequate intake or availability may need to be adjusted depending on the type of dietary data, nutrient requirement data, food composition data, whether fortification was included, and the method used to estimate inadequate intake or availability. Indicators not listed here will need to be assessed by an expert in nutritional assessment to estimate prevalence ranges for each burden category or be considered qualitatively in step 5. Implied nutrient gap burden scores should be assigned a number as follows: negligible (0), low (1), moderate (2), or high burden (3).

### Step 3: Assign metadata weights and calculate weight scores

Not all data points are equally valuable or robust. The most recent, representative, and robust data should be weighted more heavily when assessing nutrient gaps. This step describes a systematic process for attributing different weights to each qualifying data point by applying a unique weight to each metadata category specified in Table 2. For this step, exclude data points that have an evidence type of *other*, for which data collection was > 10 years ago, and for an age and sex group that excludes and is very different from the age and sex group of interest (eg, data on women of reproductive age should typically be excluded if assessing nutrient gaps for young children).

For each nutrient-specific data point meeting these specified inclusion criteria, assign weights for each of the 5 metadata categories according to Table 2: evidence type, geographic representation, recency of data collection, age and sex representation, and sample size. Among evidence types, biochemical, clinical, and functional markers have the highest weight (5), whereas nutrient adequacy of national food supplies and nutrient-informative food-group intake of individuals or households have the lowest weight (1). If metadata to assign a weight are missing, use the lowest weight for that category. If the population of interest is young children, it is recommended to change the weight for the evidence type, nutrient adequacy of household diets, to 1 rather than 2, to further penalize the lack of age representation beyond what is capable in the age and sex representation category.

Calculate weight scores (*W_s_*) for each qualifying data point using the formula, *W_s_* = *E_w_*(*G_w_ + R_w_ + A_w_ + S_w_*), where *E_w_* is the evidence type weight, *G_w_* is the geographic representation weight, *R_w_* is the recency of data collection weight, *A_w_* is the age and sex representation weight, and *S_w_* is the sample size weight. The formula multiplies the evidence type weight by the other 4 metadata categories to give more importance to data points from more robust evidence. The maximum score is 100, the minimum score 4.

### Step 4: Calculate quantitative nutrient gap burden scores

After calculating weight scores for each qualifying data point, calculate a quantitative nutrient gap burden score for each nutrient, on the basis of the implied nutrient gap burden scores from step 2 and the corresponding weight scores from step 3. To calculate the quantitative nutrient gap burden score for each nutrient, use the weighted mean of the implied nutrient gap burden score, where the weights are the weight scores—that is, multiply each implied nutrient gap burden score by its weight, sum those values, and divide by the sum of the weights.

### Step 5: Assign qualitative nutrient gap burden ratings

Initial qualitative nutrient gap burden ratings should be attributed to the quantitative nutrient gap burden scores calculated in step 4 as follows: negligible (< 0.5), low (0.5–1.49), moderate (1.50–2.49), or high (≥ 2.5). Two or more experts should review these ratings in conjunction with all available evidence, which may include data points excluded from the quantitative nutrient gap burden score and information on inflammation burden, seasonality, and temporal trends of relevant indicators. Data points excluded from the quantitative nutrient gap burden score can prove particularly valuable for adjusting quantitative nutrient gap burden scores that are based on only 1 or 2 data points with low weight scores. Temporal trends can help identify if and to what extent nutrient deficiency, intake, or availability has changed over time, which can help complement older estimates or borderline ratings, although apparent trends may also be due to methodological issues across data points. Any changes to the initial qualitative nutrient gap burden ratings must be documented to provide clear justification for the change.

### Step 6: Rate the certainty of available evidence

The recency, representation, and robustness of the available data discussed in step 3 also influence the certainty of the nutrient gap burden estimates. An initial *certainty-of-evidence* rating of low, moderate, or high should be assigned to each qualitative nutrient gap burden rating, following the guidance in Table 4 and the data point weight scores calculated in step 3. This initial certainty rating, however, can be modified. Similar to step 5, ≥ 2 experts should consider the data points that were excluded from the weight score calculation in step 3 and the general agreement or disagreement between data points. Any deviation from the initial rating should be accompanied by a clear justification for the change.

**Table 4:** Suggested certainty-of-evidence rating criteria

Low	Moderate	High
≥ 1 qualifying data point meeting minimum inclusion criteria in step 1^a^	≥ 1 data point with a weight score^b^ of 51–80 and no disagreements^c^ with any data point with a weight score > 25 Or ≥ 2 data points with a weight score 25–50 and no disagreements with any data point with a weight score > 25 Or ≥ 3 data points meeting minimum criteria thresholds, 1 of which whose weight score is ≥ 15, and no disagreements with any rating	≥ 1 data point with a weight score > 80 and no disagreements with any data point with a weight score > 50 Or ≥ 2 data points with a weight score > 50 and no disagreements with any data point with a weight score > 50

a Exclusion criteria include data collection > 20 years ago, sample size < 50, geographic representation < 10% of the total population of the geographic area of interest, and highly unhealthy participants (eg, hospitalized patients).

b Weight scores are calculated in step 3.

c A disagreement is a different implied nutrient gap burden score from step 2.

### Step 7: Qualitatively validate ratings with experts and/or local stakeholders

Depending on the individual or team involved in completing steps 1–6, consider validating qualitative nutrient gap burden ratings and certainty of evidence ratings with additional experts and/or local stakeholders. The evidence summary generated in step 1, along with the ratings and justifications from steps 2–6, should provide clear guidance for any additional validators. Additional experts and local stakeholders may have more knowledge of how to interpret implied nutrient gap burdens rated in step 2, awareness of relevant contextual information about the population of interest, awareness of methodological or data quality issues for evidence sources that are not documented in published reports, or objectivity from not having reviewed the evidence sources and data points in depth.

### Step 8: Produce guidance document for decision makers

After finalizing and qualitatively validating all ratings, it is essential to produce a guidance document that is easy to interpret for nontechnical decision makers. Nutrients with a final qualitative nutrient gap burden rating of moderate or high *and* a certainty-of-evidence rating of moderate or high should be considered high-priority nutrient gaps for policy, programs, and research aimed at improving nutrient intake. Consider identifying locally available foods dense in nutrients that can fill high-priority nutrient gaps and exploring other strategies for filling these gaps, such as biofortification, fortification, and supplementation. Nutrients with a final qualitative nutrient gap burden rating of moderate or high *and* a certainty-of-evidence rating of low should be considered potential nutrient gaps and prioritized for new data collection and evidence generation. It is important to present findings and policy implications in a clear and simple guidance document that is accessible to nontechnical audiences, including high-level decision makers, but also includes an appendix with detailed rating justifications for technical audiences.

## CONCLUSION

Here we have proposed and described a new method called CONGA for identifying nutrient gaps and evidence gaps by reviewing multiple evidence sources based on biomarker and/or dietary data. To enable the use of various types of evidence to assess nutrient gaps, 5 main evidence types were defined and their strengths and limitations discussed. We then described how to find and compile the evidence to facilitate interpretation and synthesis. Finally, guidelines were provided on how to semiquantitatively rate the burden of nutrient gaps and certainty of evidence, considering all relevant evidence, and how to present results and their implications.

The CONGA has unique strengths and utility. A CONGA estimates the burden of nutrient gaps to provide insight into their public health significance, as well as the certainty of estimates, and identifies nutrients that need additional data collection and evidence generation. Comprehensive Nutrient Gap Assessment uses all relevant evidence types available, which often tell different stories, allowing for a more complete understanding of nutrient gaps. A CONGA can be conducted relatively quickly and affordably and does not require access to raw data. The simplicity of the qualitative rating categories also makes the results easily interpretable by nontechnical decision makers. Finally, a CONGA can be updated with minimal effort and resources when new evidence becomes available, because all prior data would have already been summarized and categorized in an editable spreadsheet, allowing for easy tracking of progress over time. A CONGA does not replace the need for additional and improved biomarker and dietary intake data and modeling studies. In fact, with a preponderance of low-quality evidence for many micronutrients, CONGA highlights the need for additional high-quality data. Robust, nationally representative data and analysis on nutrient gaps are urgently needed to guide action on how to improve diets and will help further improve the quality of CONGA and other related analyses.

The CONGA has important limitations. Despite using a wide array of available evidence, there are still contexts in which the breadth or depth of available data significantly limits the certainty with which nutrient gap burdens can be rated. Although this process can shed light on evidence gaps, it cannot overcome them. In addition, the lack of established guidance on the public health significance of prevalence ranges for biomarkers and dietary nutrient gaps requires some degree of estimation and subjectivity in ratings. Thus, the quality of any CONGA may be biased or limited by the knowledge of the individual(s) involved. However, CONGA provides clear guidelines for ratings, documentation, and qualitative validation to provide transparency and accountability. Including a broad set of nutritional and public health expertise and local knowledge will improve the quality and acceptance of the findings.

Other methods exist for collating and assessing a wide range of data sources, with or without primary or secondary data analysis, in an effort to better guide policy and programming decisions on diets.^36^ For example, the Fill the Nutrient Gap exercise designed and implemented by the World Food Programme uses both primary and secondary data collection and triangulates findings on dietary practices and quality.^37^ Fill the Nutrient Gap reports, where available, are recommended to be included in the qualitative evidence reviewed in a CONGA, because they provide a comprehensive look at the environment within which observed diets are shaped. However, the Fill the Nutrient Gap exercise itself is designed as a systems-focused situation analysis of barriers to consuming an adequately nutritious diet. The process takes a comprehensive look into the enabling environment, stakeholder engagement, and food system and cost barriers, and uses modeling to identify potential platforms for increased nutrient intake.^37^ This assessment of how systems are succeeding or failing to provide access to nutritious diets is valuable for design of national policies and strategies but also requires substantial human and financial resources. Moreover, the Fill the Nutrient Gap exercise provides no estimates of nutrient gaps, their health impacts, or the certainty of evidence reviewed.

Insight into nutrient gaps and evidence gaps is essential for informing recommendations on how to improve diets, and thus the nutritional status, of a population. Evidence indicative of the burden of nutrient deficiencies or inadequate nutrient intake or availability are frequently available in countries yet often misinterpreted or underused in decision-making. The CONGA provides a systematic approach to use this evidence to aid decision makers in understanding what policies, programs, and research are required to improve diets. Furthermore, CONGA findings can be used to inform follow-up analyses to explore the primary causes of nutrient gaps, such as inadequate availability, accessibility, affordability, desirability, or knowledge of nutritious foods rich in priority nutrients. For example, authors of 2 papers in this *Nutrition Reviews* supplement have assessed the affordability of locally available and culturally appropriate foods that can fill high-priority nutrient gaps in countries in Eastern and Southern Africa and South Asia.^38^^,^^39^

## Supplementary Material

nuaa140_Supplementary_Data
