# Supplementary material for: Comprehensive Nutrient Gap Assessment (CONGA): A method for identifying the public health significance of nutrient gaps
Source: Nutr Rev. 2021 Mar 8;79(Suppl 1):4–15. doi: 10.1093/nutrit/nuaa140 (PMC7947985; doi:10.1093/nutrit/nuaa140)
Supplement: nuaa140_Supplementary_Data [file nuaa140_supplementary_data.zip › Figure S1.docx]

**
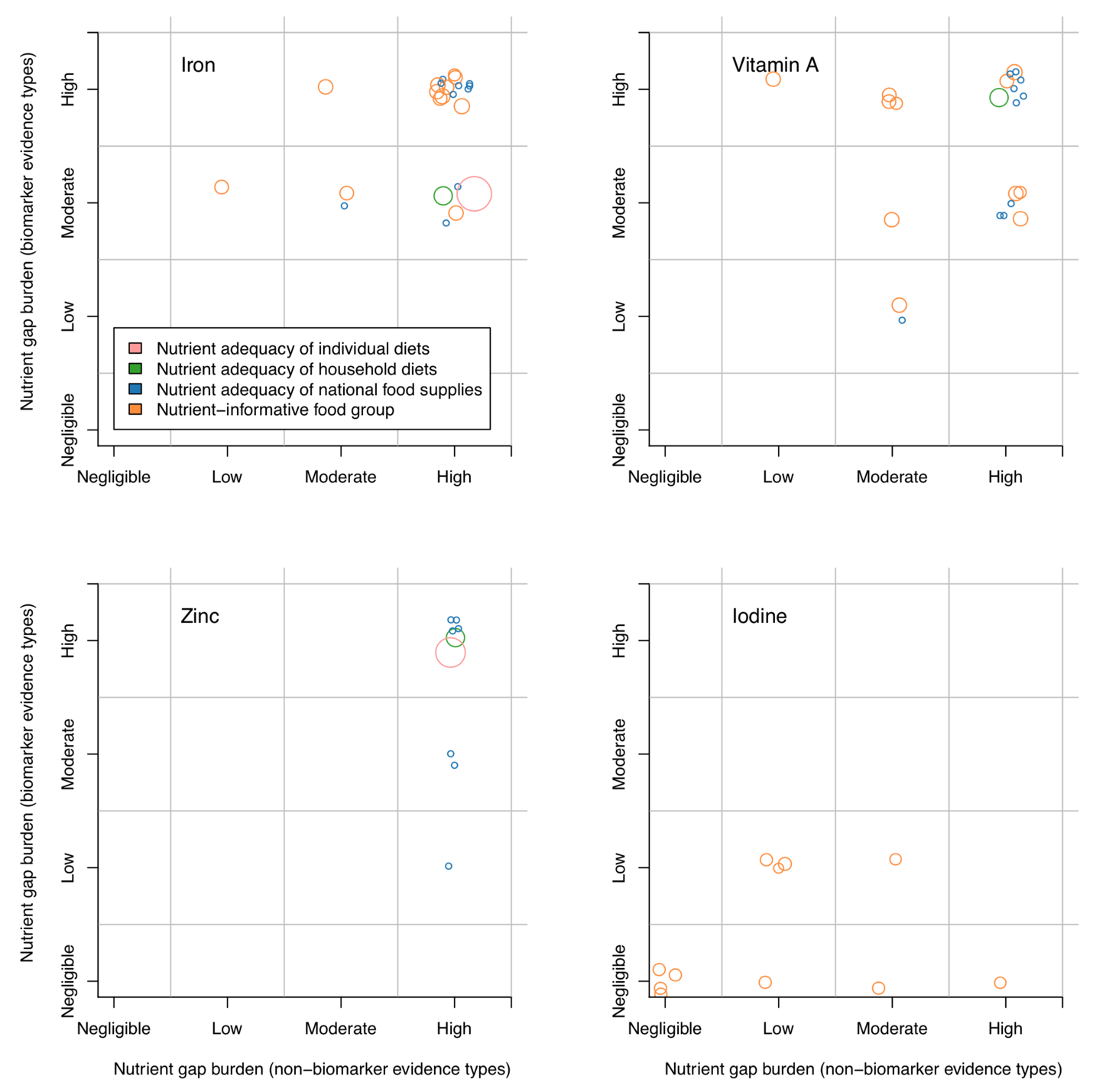
**

**Figure S1.** Implied micronutrient gap burden ratings of biomarker data points compared with implied micronutrient gap burden ratings of non-biomarker data points for children 6–23 months in 14 countries in Eastern and Southern Africa and South Asia. Circles represent each non-biomarker data point that qualified for the quantitative burden score and had a corresponding qualifying biomarker data point from the same country. The size of the circle represents the weight score of that data point.
